# Neurosyphilis Presenting as Rapid Cognitive Decline With Demonstrated Improvement Posttreatment: A Case Report

**DOI:** 10.1155/crdi/1161141

**Published:** 2026-07-12

**Authors:** James Smith, Michael Angputra, Roger Ho

**Affiliations:** ^1^ Geriatrics Department, Western Health, Footscray, Melbourne, Victoria, Australia, westernhealth.org.au

**Keywords:** cognitive impairment, dementia, neurocognitive disorder, neurosyphilis, syphilis

## Abstract

Syphilis has become an increasing public health concern in recent times, with rising incidence globally. It is often referred to as the ‘great imitator’ due to its diverse clinical presentations across multiple stages. Neurosyphilis, a tertiary manifestation of *Treponema pallidum* infection, can present with neuropsychiatric features including rapid cognitive decline. It remains an important but potentially overlooked cause of cognitive impairment (CI). However, few cases document objective cognitive and functional improvement following treatment, especially within a short time frame. We describe the case of a 67‐year‐old man with rapid cognitive decline, displaying impairments in various cognitive domains: learning and memory, attention and executive functioning. The presence of Argyll–Robertson pupils combined with positive serological and cerebrospinal fluid testing confirmed a diagnosis of neurosyphilis. Following treatment with intravenous penicillin G, serial cognitive assessment demonstrated objective improvement in his cognition and functioning within 1 month of treatment. Our observation of such an improvement in neurosyphilis‐driven CI in this time frame is a finding not commonly documented in associated literature. This case highlights the importance of thorough history taking, including a sexual history, alongside physical examination in diagnosing neurosyphilis. Additionally, it supports the importance of considering neurosyphilis when investigating patients with unexplained cognitive decline and suggests there is a degree of reversibility when treated promptly. Further research is needed to better characterise the treatability of neurosyphilis‐related CI.

## 1. Introduction

Syphilis is a sexually transmitted infection (STI) caused by the bacterium *Treponema pallidum* (*T. pallidum*). Its global incidence has been increasing in recent years, particularly amongst men who have sex with men (MSM) [1]. Recent data also show increasing rates amongst women and heterosexual men [2]. International epidemiological data show significantly rising prevalence rates in multiple continents over the past 10 years [3]. This reflects a significant public health issue that has caught the attention of governments worldwide, prompting various national response plans [4, 5].

Syphilis is often referred to as ‘The Great Imitator’ given its varied clinical presentation. It can progress through four separate stages if left untreated: primary, secondary, latent and tertiary. Importantly, evidence of tertiary syphilis can manifest months to decades after initial exposure [6]. It can be subcategorised into gummatous syphilis, cardiovascular syphilis or neurosyphilis and can develop in up to 40% of the untreated cases [6].

Neurosyphilis refers to a syphilis infection affecting the central nervous system (CNS). It can occur at any stage following infection from *T. pallidum*. The diagnosis of neurosyphilis requires a combination of positive serum testing, history, physical examination findings and positive cerebrospinal fluid (CSF) treponemal or nontreponemal tests. Neurosyphilis can be categorised into early, intermediate or late disease [6]:

### 1.1. Early Neurosyphilis

- Asymptomatic neurosyphilis is the most common type [7].
- Meningeal neurosyphilis: Diffuse infiltration of disease into the meninges, presenting with features such as headache, nausea, cranial nerve defects and meningism [7].

### 1.2. Intermediate Neurosyphilis

- Meningovascular neurosyphilis: Meningeal inflammation with endarteritis, it can present similarly to meningeal neurosyphilis, but most commonly as a middle cerebral artery (MCA) stroke in a younger adult [7].

### 1.3. Late Neurosyphilis

- Syphilitic (general) paresis: Chronic meningoencephalitis, which can present with cognitive impairment (CI), personality changes or psychiatric disturbance [7].
- Tabes dorsalis: Destruction of the dorsal columns, presenting with features such as neuropathic pain, ataxia and pupillary abnormalities [7].

Given the varied and often nonspecific presentations of neurosyphilis, it is a diagnosis that may commonly be overlooked, with some research suggesting presentations can be more commonly atypical [8]. Neurosyphilis is known to cause CI [9]. As CI is a common presentation in hospitals, there is a risk that cases of neurosyphilis may be misdiagnosed, resulting in the missed opportunity to potentially reverse a cognitive decline. At present, literature documents CI as a well‐known symptom of neurosyphilis, but there is limited information that details its level of reversibility [9].

In this article, we present the case report of a 67‐year‐old gentleman with progressive CI, who was diagnosed with and treated for neurosyphilis. He demonstrated objective improvement in his cognition and functioning shortly after treatment.

## 2. Case Report

A 67‐year‐old previously independent man with no significant past medical history had multiple presentations to the emergency department after being catheterised for acute urine retention and inability to self‐manage the catheter at home. A subsequent community geriatrician review raised concern for CI with an initial Rowland Universal Dementia Assessment Scale score of 22/30. He was then admitted to hospital following a fall at home. Collateral history revealed a 6‐month history of cognitive decline, predominated by amnestic features, repetitive conversations and impaired learning. The latter 3 months included impaired executive functioning, increased irritability, tangentiality and disorientation. Additionally, there was evidence of self‐neglect and an inability to cope at home independently. History taking also revealed binge‐pattern alcohol consumption which was initially thought to explain his CI.

Further careful history taking identified the patient as part of the MSM group with no previous STI screening. Serological testing revealed a positive rapid plasma reagin (RPR) at a titre of 1:128. The diagnosis of syphilis was confirmed by a reactive treponemal pallidum particle agglutination (TPPA) test. At this time, other serological results including inflammatory markers, electrolytes, vitamin B12, thyroid function and liver function tests were all unremarkable. Lumbar puncture yielded clear CSF with a white cell count of 14 × 10^6^/L (reference range < 3.6–11 × 10^6^/L), glucose concentration of 3.2 mmol/L (reference range 2.0–3.9 mmol/L), protein concentration of 0.65 g/L (reference range 0.15–0.45 g/L) and no growth on culture. CSF treponemal testing showed reactive total antibodies on chemiluminescence assay (CLIA), a RPR titre of 1:4, reactive TPPA and positive fluorescent treponemal antibody absorption (FTA‐ABS). Screening for other STIs including serum human immunodeficiency virus antibody/antigen, urinary Chlamydia trachomatis and Neisseria gonorrhoea antigens were negative. Magnetic resonance imaging (MRI) of the brain showed mild periventricular and subcortical chronic small vessel ischaemia without evidence of leptomeningeal enhancement, dural thickening or any significant temporal/hippocampal atrophy.

Physical examination revealed the presence of 1‐mm pupils bilaterally that did not react to light, but accommodated appropriately, consistent with Argyll–Robertson pupils, and hyperreflexia of both achilles tendons. No other physical stigmata of syphilis infection were observed. After review by the infectious diseases team, a diagnosis of neurosyphilis was made.

The patient was treated with 15 days of intravenous (IV) aqueous crystalline penicillin G dosed at three millions units, every 4 h. This is in accordance with current international treatment guidelines for neurosyphilis [10]. Estimated glomerular filtration rate at the time was 80 mL/min/1.73 m^2^, and no contraindications to penicillin were present. An acute delirious episode occurred within 9 h of therapy initiation, with visual hallucinations present, which resolved within 24 h. Though Jarisch–Herxheimer reactions (JHRs) are well recognised after the treatment of spirochaetal infections, their neuropsychiatric manifestations are not so well documented within literature [11]. We suspect the timing of this episode could represent a CNS form of a JHR. Whilst the proximity of such a reaction to treatment initiation has a noteworthy temporal relationship, true causality cannot be confirmed in this case. Cognitive testing was performed 3 days after the initiation of antibiotics, yielding an Addenbrooke’s Cognitive Examination Revised (ACE‐R) score of 68/100 with particular impairment in memory and attention/orientation. A further ACE‐R was performed 28 days later (16 days after finishing the antibiotic course) with an improved score of 77/100. There was a significant improvement in his attention score (11/18–17/18) but a persistently poor score in memory (10/26–9/26). The absence of delirium at the time of the initial ACE‐R may suggest the abnormal score was largely a reflection of his neurosyphilis‐induced CI. Thus, the score improvement posttreatment may represent a clinically significant finding, which suggests a degree of disease reversibility. The patient remained admitted under subacute geriatric services for a further 2 months, displaying more consistently settled behaviour, reduced tangentiality and more consistent orientation to time and place. In addition to the cognitive improvement, the patient made improvements in functionality after treatment. Before treatment, he was unable to perform personal activities of daily living without supervision and support from staff. After treatment, he was able to perform all personal activities of daily living independently. A follow‐up phone call to his family 2 years later was suggestive of further subjective improvement in his cognition; however, the patient had declined further cognitive testing.

## 3. Discussion

Neurosyphilis is an important but likely underrecognised cause of CI, particularly in the context of rising syphilis incidence globally [1–3]. It is important to remember that symptoms of neurosyphilis can present from months to decades after infection and overlap with other primary psychiatric or other neurodegenerative disorders [6, 12].

Our patient in this case presented with cognitive decline thought to be secondary to neurosyphilis. Without careful history taking or consideration of performing syphilis serology testing, this diagnosis may have been missed and the patient may not have had the opportunity to make cognitive improvement. Despite the absence of radiological features seen in neurosyphilis in this case, neuroimaging is supportive, not diagnostic; neurosyphilis remains a clinical diagnosis, supported by serological and CSF testing [13]. Collateral history from the patient’s family suggested previous alcohol excess which initially led to an underappreciation of his CI. This potentially led to an anchoring bias by the treating team before further history was taken and the correct diagnosis was reached.

Previous reports within the literature have established CI as a feature of neurosyphilis; meanwhile, our case documents an objective improvement in cognition and functioning within 1 month attributed to sufficient treatment as suggested by the pre and post ACE‐R scores of 68/100 and 77/100, respectively [14, 15]. Further published cases that explore serial improvements in cognitive function have been documented but are limited in comparison [16].

These findings suggest that CI attributed to neurosyphilis is treatable, especially if diagnosed and managed promptly, at least in the short term. Our case does not provide objective evidence of long‐term cognitive recovery, limited by the loss of patient follow‐up.

Furthermore, given the incidence of syphilis is rising internationally, similar cases to ours may become more prevalent [1–3]. It is important to raise awareness amongst clinicians of considering a diagnosis of neurosyphilis when faced with unexplained CI, especially if other risk factors are identified. However, there are important considerations to lowering the syphilis screening threshold, particularly patient acceptability and perceived stigma, which can negatively impact treatment adherence and overall quality of life [17]. It is unclear how practical or cost‐effective it would be to routinely screen for syphilis in all CI patients, but more targeted screening strategies could lead to earlier diagnoses.

The JHR is a well‐described phenomenon, usually seen in early stage syphilis. It is a transient, systemic reaction thought to be caused by rapid lipoprotein release from spirochaetal bacterium after initial exposure to antibiotics [18]. However, it is less commonly documented in cases of neurosyphilis, and its manifestation in tertiary infection is not as well characterised. We suspect the acute exacerbation in this patient’s neuropsychiatric symptoms reflects a JHR given his disease burden was focused within the CNS. One prospective cohort study from 2024 described a 9% incidence of JHR in treated patients with neurosyphilis within their cohort with an average peak worsening of symptoms at eight hours postinitial antibiotic [11]. Agitation and confusion was only described in 10% of these patients with JHR, suggesting it is a less common manifestation [11]. Our suggestion of JHR in this case, which presented with acute agitation, is an important and possibly underrecognised finding in these patients, suggesting the need for further research into how JHR can manifest in neurosyphilis.

## 4. Conclusion

Neurosyphilis remains an important and potentially underdiagnosed cause of CI, with diverse neuropsychiatric manifestations. This case suggests that sufficient treatment can lead to potential improvements in cognition and functioning. There is a need for further research into the treatment of neurosyphilis‐related CI, and additional case reports which measure serial cognitive assessments after treatment would provide valuable evidence to our theory. We suggest that interval cognitive assessment pre and posttreatment is a vital part of monitoring progression in patients who have neurosyphilis with CI.

## Funding

No specific funding was supplied for the purpose of this project.

## Consent

All the patients allowed personal data processing, and informed consent was obtained from all individual participants included in the study.

## Conflicts of Interest

The authors declare no conflicts of interest.

## Data Availability

The data that support the findings of this study are available from the corresponding author upon reasonable request.
